# Measuring Mental Effort for Creating Mobile Data Collection Applications

**DOI:** 10.3390/ijerph17051649

**Published:** 2020-03-03

**Authors:** Johannes Schobel, Thomas Probst, Manfred Reichert, Winfried Schlee, Marc Schickler, Hans A. Kestler, Rüdiger Pryss

**Affiliations:** 1 Institute of Medical Systems Biology, Ulm University, 89069 Ulm, Germany; 2 Institute of Databases and Information Systems, Ulm University, 89069 Ulm, Germany; 3 Department for Psychotherapy and Biopsychosocial Health, Danube University Krems, 3500 Krems, Austria; 4 Department of Psychiatry and Psychotherapy, University of Regensburg, 93053 Regensburg, Germany; 5 Institute of Clinical Epidemiology and Biometry, University of Würzburg, 97070 Würzburg, Germany

**Keywords:** data collection, smart mobile devices, end-user programming, mental effort, usability study

## Abstract

To deal with drawbacks of paper-based data collection procedures, the QuestionSys approach empowers researchers with none or little programming knowledge to flexibly configure mobile data collection applications on demand. The mobile application approach of QuestionSys mainly pursues the goal to mitigate existing drawbacks of paper-based collection procedures in mHealth scenarios. Importantly, researchers shall be enabled to gather data in an efficient way. To evaluate the applicability of QuestionSys, several studies have been carried out to measure the efforts when using the framework in practice. In this work, the results of a study that investigated psychological insights on the required mental effort to configure the mobile applications are presented. Specifically, the mental effort for creating data collection instruments is validated in a study with N=80 participants across two sessions. Thereby, participants were categorized into novices and experts based on prior knowledge on process modeling, which is a fundamental pillar of the developed approach. Each participant modeled 10 instruments during the course of the study, while concurrently several performance measures are assessed (e.g., time needed or errors). The results of these measures are then compared to the self-reported mental effort with respect to the tasks that had to be modeled. On one hand, the obtained results reveal a strong correlation between mental effort and performance measures. On the other, the self-reported mental effort decreased significantly over the course of the study, and therefore had a positive impact on measured performance metrics. Altogether, this study indicates that novices with no prior knowledge gain enough experience over the short amount of time to successfully model data collection instruments on their own. Therefore, QuestionSys is a helpful instrument to properly deal with large-scale data collection scenarios like clinical trials.

## 1. Introduction

A multitude of domains, such as healthcare, psychology, and social sciences, still heavily relies on paper-based instruments (e.g., self-report questionnaires [1]) to collect data in various situations and for different purposes. Despite numerous existing drawbacks, traditional data collection procedures are still widely used. However, when dealing with large-scale studies like clinical trials, paper-based procedures are time-consuming and error-prone. To deal with such shortcomings, many web-based questionnaire applications (e.g., Qualtrics or SmartSurvey) have been developed, allowing researchers to create online questionnaires themselves. In this context, the authors of [2] estimate that 50–60% of the costs related to collecting, transferring, and processing data could be saved when using digital instruments instead of paper-based ones. Furthermore, the authors of [3] showed that electronic versions do not affect psychometric properties of questionnaires, but, in turn, enhance the overall quality of the data collected [4]. Finally, digital versions allow for more comprehensive datasets [5] through the application of automatic validation rules or the opportunity to add context information like the time or a location when using smartphones [6]. In addition, the built-in sensors of smartphones can be used to gather even more valuable data, e.g., by measuring vital parameters during an interview [7]. Altogether, digital implementations of paper-based instruments are increasingly demanded in many scenarios [8]. Despite the aforementioned benefits, the offered web-based questionnaires are often not suitable in various scenarios. For example, web browsers may not be able to properly collect data from required sensors (e.g., pulse sensors). *Smart mobile devices* (e.g., smartphones, tablets, etc.), in turn, may provide the required features in order to enable researchers to collect the data in the demanded scenarios, like healthcare [9,10,11]. In addition, the collection procedure can be improved to gather large amounts of data in a rather short time, which was already proven in existing studies (see, e.g., [12] or [13]).

In the larger context of mobile data collection, platforms like *TrackYourTinnitus* [14], *Manage My Pain* [15], or *PsychLog* [16] already rely on smart mobile applications to collect huge amounts of patient data in a convenient fashion. Other existing works apply such novel techniques in smaller application scenarios [17,18,19]. Moreover, the authors of [20] summarize results from a systematic literature review regarding the use of (smart) mobile applications in healthcare domains. However, when investigating the development processes used by these approaches, several issues can be observed. The authors of [21], for example, report problems in the communication and interaction between researchers and IT experts as well as huge financial costs for developing sophisticated mobile applications. Furthermore, the authors of [22] report challenges related to the deployment of applications running on smart mobile devices in clinical scenarios (e.g., security concerns). Relying purely on smart mobile devices when collecting data may exclude specific participant groups (e.g., elderly) [23]. Furthermore, providing respective mobile applications for only one mobile platform (e.g., Android or iOS) may result in biased samples as the mobile platforms users may differ regarding various aspects such as income, age, or education [24].

Taking the mentioned findings into account, the *QuestionSys* framework was developed to mitigate the drawbacks of existing approaches on one hand, while exploiting the capabilities of modern smartphones on the other. The framework, in turn, applies *end-user programming* techniques to ease the *process of modeling* data collection instruments. Thereby, it offers an intuitive configurator component that allows researchers to flexibly create their instruments in a graphical manner. More specifically, QuestionSys applies its own modeling notation that is, on the one hand, influenced by BPMN 2.0 (Business Process Model and Notation), but reduces overall complexity on the other [25]. In a pilot study [26] with 44 participants, we already revealed promising results with respect to the modeling process of such instruments. It could be shown that prior process modeling experience does not influence performance measures (such as time or errors) when working with the developed QuestionSys configurator. Based on these insights, a larger study with a more sophisticated design was performed. The manuscript at hand presents results from this study. More specifically, the following research questions (RQs) were addressed with novices (with no prior experience in process modeling) and experts (with experience in process modeling):

**RQ 1:** How does the self-reported mental effort change when modeling several data collection instruments?

**RQ 2:** How are the performance measures of novices and experts compared to the self-reported mental effort at each data collection instrument?

**RQ 3:** How are the performance measures of novices and experts compared to the self-reported mental effort across all data collection instruments?

## 2. Material and Methods

In this section, some background information on the QuestionSys framework is presented. Further, the methods used to collect the study data are described in detail. The Ethics Committee of Ulm University approved all materials and methods (#196/17); thus, the study was carried out according to the approved guidelines. In particular, all participants obtained and approved the written informed consent.

### 2.1. QuestionSys Framework Background Information

Over the last years, the authors were able to support researchers in collecting data in large-scale scenarios by supporting them with many specifically tailored mobile applications (see Table 1). By realizing these mobile data collection applications, domain-specific requirements could be elaborated [27].

**Figure 1:**
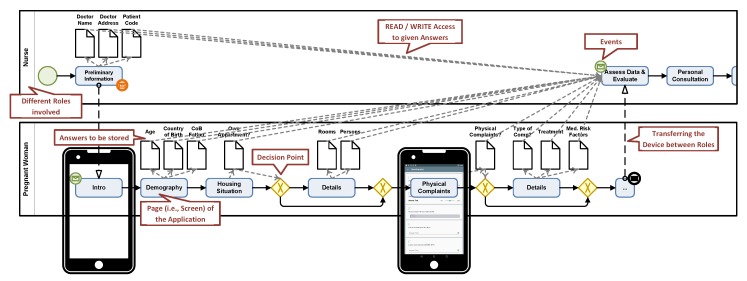
A data collection instrument represented as BPMN (Business Process Model and Notation) 2.0 Model.

Although the developed mobile applications were sufficient to support researchers in their studies, most of them demanded additional features (e.g., record the audio or measure vital parameters during the interview) very shortly after a study started. In this context, the developments of specifically tailored applications for a particular scenario turned out to be time-consuming, especially when trying to keep pace with the short release cycles of the mobile operating system vendors (e.g., Google and Apple). Besides, developing easy-to-understand user interfaces is very challenging, depending on the respective application scenario [33]. Furthermore, we revealed communication issues between researchers and application developers that must be tackled, as described in [21]. More specifically, we realized that both parties used different languages (i.e., wording, or (graphical) notations) for the same aspects. To relieve developers from constantly adjusting or enriching existing solutions on one hand, and empowering researchers to develop their own applications on the other, the QuestionSys approach is proposed. It articulates data collection instruments in terms of *process models* (see Figure 1). These models, in turn, may then be enacted based on a well-defined execution semantics directly on smart mobile devices, such as smartphones or tablets. However, other approaches exist that enable researchers to collect large amount of data using mobile applications as well. The authors of [16], for example, present a platform that allows for collecting information on mental health issues. The platform itself comprises two modules: The *Survey Creator* allows for creating customizable data collection procedures (e.g., questionnaires), whereas the *Sensing Module* renders this procedure on a smart mobile device to collect the data. The authors of [34] discuss an approach that relies on *WordPress* and *iBuildApp* to create a platform supporting students from clinical psychiatry. Although the developed platform mainly focuses on information representation (e.g., provide learning material or treatment guidelines), it is possible to create individual questionnaires evaluating the study progress of participants. The authors of [35] present a smart mobile application to capture deviations from standardized healthcare processes. Thereby, medical staff are advised to fill in questionnaires related to the performed examinations to provide valuable feedback to the treatment. In [36], a process-oriented approach for creating mobile business applications is presented. The authors describe how their used process models can be transformed to application code for different mobile platforms (e.g., Android and iOS). The application code, in turn, is the basis for the mobile application logic. The overall procedure, however, results in a mobile application for which the logic is hard-coded. As a consequence, many promising key features from process technology research are less exploited. Other projects, like *MagPi* or *MovisensXS*, also provide configurators using simple web forms allowing end-users to create applications for data collection. Compared to the presented approach, these applications are limited regarding the provided instrument features. More specifically, advanced features like a *navigation logic* (e.g., influence the further course of the instrument based on already given answers) are not provided.

In general, the use of graphical process modeling notations (i.e., BPMN 2.0 or EPC) provides manifold features, ranging from a well-defined semantic to execute processes correctly, to features that are required in the context of enterprises [37]. On the other hand, the variety of provided elements has proven to hinder researchers with no (or little) process modeling experience in *creating* a data collection instrument by themselves. For example, various elements needed in the context of complex business processes are not required to describe data collection instruments (e.g., time constraints or relationships between involved business partners). In particular, managing the *data flow* within an instrument revealed to be difficult due to several reasons: First, the type of data (i.e., numeric, alphanumeric, etc.) has to be defined properly. Second, process activities can only read data if preceding process activities have generated (i.e., write) the respective data. Third, gateways consume (i.e., read) such data to properly control the flow of the process model during the execution if needed. Modeling such complex data flow, in general, represents a difficult endeavor for researchers with no or little process modeling knowledge.

To allow untrained staff to use such a powerful modeling approach to graphically develop data collection instruments, so-called *end-user programming* techniques were evaluated [38]. The authors of [39] discuss the importance of empowering non-developers by providing sophisticated tools. The authors of [40], in turn, report a divergence between trained software developers and self-reported end-user programmers, thus strengthening the feasibility of such approaches. In general, in a multitude of studies, such approaches have proven their applicability in supporting non-programmers to achieve certain goals. For example, the authors of [41,42] present a graphical programming language, which was specifically tailored for pupils. Thereby, instructions are represented as colored blocks that may be combined to *write* a software application. Teachers reported that the simplified representation significantly improved the pupils understanding of program code (especially on deeply-nested code fragments) [43]. The authors of [44] presents a user interface-centric approach for web service composition. Thereby, a visual editor allows for the mapping of structured data from files (e.g., spreadsheets) to user interface elements (e.g., lists or tables). A conducted study with 36 participants proves the applicability of the discussed approach. Finally, the authors of [45] present a live programming environment that allow non-programmers to transform spreadsheet-like data into web applications. Thereby, local files and web services may be combined using a graphical editor. However, the authors rely on formulas known from common spreadsheet applications (e.g., Microsoft Excel) to ease the mental effort required to create such applications. Note that other studies exist, which are also proving the feasibility of such (graphical) end-user programming approaches in specific application domains, in which untrained staff needs to be properly supported.

### 2.2. Study Procedure

For the study presented in this paper, the recruited participants had to model several data collection instruments using only the provided configurator component. Over the course of 2 sessions, 5 data collection instruments had to be modeled at each session, with one week respite between Session 1 and Session 2. To quickly react to emerging problems, a computer pool at Ulm University was chosen as a controlled environment for this study. Thereby, 20 workstations (each comparable in hardware, like RAM or CPU cores) were carefully prepared for each session. For example, the configurator component was re-installed for each round, or respective task descriptions and consent forms were newly placed beside the workstations.

**Figure 2:**
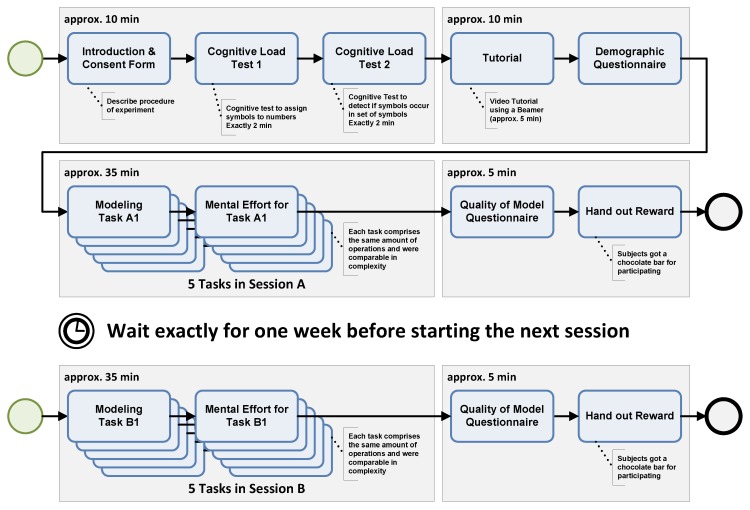
Study design.

Figure 2 introduces the study design: When welcoming the participants, they were introduced by explaining the goal of the study as well as the overall course. Then, participants had to process two tests measuring their cognitive load when working under pressure (2 min each). Next, they watched a short screencast (~5 min playtime) that introduces the most important aspects and feature of the configurator component that should be analyzed. Finally, participants were asked to fill in a short questionnaire assessing demographic information about their person. Up to this point, participants were allowed to ask questions regarding the configurator or the study itself. For the main part of the study, participants were given five tasks to be processed (i.e., data collection instruments to be modeled; see Table 2). Thereby, they were only allowed to use the provided configurator component. After modeling each instrument, they were asked to fill in a short questionnaire regarding their mental effort when working on respective task. Finally, they had to answer one last questionnaire asking details on the overall quality of the modeled instruments. Altogether, the first session took approximately 50 to 60 min in total (depending on the participants speed).

Session 2 started exactly after pausing for one week. Note that the collection of demographic information and the tutorial were skipped, resulting in a much shorter session. Participants only had to process five new tasks (i.e., model five new data collection instruments, see Table 2) and answer the mental effort questionnaires again. In addition, they had to give again feedback on the quality of their modeled instruments.

### 2.3. Participants

For the study, we recruited 80 participants (mainly students and research associates) from different faculties (e.g., computer science, economics, chemistry, psychology, and medicine) at Ulm University. It was ensured that almost the same number of female and male participants were recruited. Then, the participants were (1) instructed to adhere to the study design and (2) were told that they must accomplish two consecutive sessions to successfully complete the study. The material for the study (e.g., task descriptions, consent form, and questionnaires) was provided in German [46]. According to the study design, participants who answered the question *“Do you have experience in process modeling”* with *yes* were classified as *experts*. On the other, participants who answered the question with *no* were classified as *novices* for the subsequent analysis. Altogether, this resulted in 45 novices and 35 experts (80 in total). Note that only 3 out of the 80 participants did not show up for Session 2 (one novice and two experts). Research questions that require data from Session 2, therefore, were investigated with 77 participants (44 novices and 33 experts) instead of 80 (45 novices and 35 experts).

### 2.4. Configurator Component

**Figure 3:**
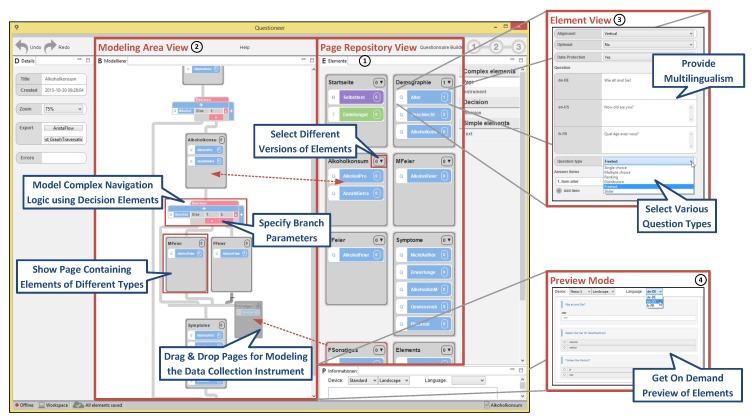
The QuestionSys configurator.

The developed configurator component applies sophisticated techniques from end-user programming and process management technology. This combined use of well-known technologies and approaches enables researchers to create mobile data collection instruments without involving any IT experts. The most important aspects of the configurator component are sketched in Figure 3 (see [47] for more details).

**(1) Page Repository View:** The page repository shows all available pages of this instrument. Selecting a page or an element (e.g., texts and questions) from a page shows a live preview (see Figure 3 ④). Furthermore, elements may be customized in the element view (see Figure 3 ③).

**(2) Modeling Area View:** Available pages from the page repository (see Figure 3 ①) may be used to model the structure of the data collection instrument. This graph-based approach allows researchers to model sophisticated navigation operations (e.g., skip pages based on already given answers) to adapt the instrument during the data collection process. The graphical editor, in turn, provides guidance for the domain experts, i.e., it does not allow to apply *wrong* operations and indicates errors within the model. Furthermore, it also provides a preview for the instrument (see Figure 3 ④).

**(3) Element View:** The element repository allows for creating and managing basic elements of a questionnaire (e.g., texts and questions). More specifically, the configurator allows handling elements in multiple languages and revisions. Furthermore, this view allows combining elements to pages by applying simple drag and drop operations.

**(4) Preview Mode:** To provide some kind of *What You See Is What You Get* (WYSIWYG) approach, the configurator component provides a preview mode. This mode, in turn, allows for simulating the instrument on a specific smart mobile device (e.g., iPhone) with a requested language (e.g., German).

The described configurator allows researchers to visually create sophisticated data collection instruments. Thus, the overall development costs and time can be significantly reduced.

For this study, the configurator component was enhanced to provide a self-contained *Study Mode* that enables specific features: First, it requires participants to enter a *code* before starting the application. This code, in turn, is used to assign all collected information to one specific participant. Second, the configurator automatically tracks performance metrics, like the *time* when a specific *operation* (e.g., adding a page to the instrument) was performed. Third, right after performing the operation, an *image* of the current state of the data collection instrument is stored on the computer, allowing to reproduce the modeling process of participants step-by-step.

### 2.5. Performance Measures

This section describes the performance metrics that are automatically assessed by the configurator component (see Section 2.4).

#### 2.5.1. Time

When participants start working on their data collection instrument, the current timestamp is added to a excel file stored in the application’s directory. After completing modeling, again, the current timestamp is captured. Based on these two values, the overall time (measured in ms) a participant needed to complete a task was calculated. Furthermore, additional timestamps are captured after performing an operation (e.g., adding a page to the instrument).

#### 2.5.2. Operations

After interacting with the instrument (e.g., add or remove a page) the performed operation and timestamp is logged to the described excel file. Furthermore, the configurator component generates an image of the current state of the model after performing this operation. All images are stored in the application’s directory.

#### 2.5.3. Errors

Unfortunately, it was not possible to automatically assess the errors in the final models of the participants. This is because multiple solutions may be correct and valid. For example, it does not depend on the order of branches in decision points, but rather on the decisions that are assigned to these branches. Therefore, all models (10 data collection instruments per participant) were evaluated manually based on the automatically generated instrument images.

### 2.6. Tutorial

We recorded a short screencast tutorial showing the most important aspects of the developed configurator component. In particular, the graphical modeling editor was introduced by creating one simple data collection instrument. As it was not possible to play audio in our controlled environment (computer pool at Ulm University), we added small annotations in post-production. This video was presented to the participants on their monitors.

### 2.7. Tasks

During the course of the study, participants were asked to create 10 data collection instruments (5 at Session 1 and 5 at Session 2). Each task to perform was handed out as a textual representation describing the scenario (e.g., collect patient information for an upcoming surgery) and the structure of the data collection instrument to be modeled. Thematically, the models to be created were selected from various domains, ranging from a *travel expense report* up to a *questionnaire for healthcare support* (see Table 2). All participants had to process all described tasks and worked on the tasks in the same order.

All tasks to be modeled were comparable regarding their textual description, the complexity of the resulting instrument, and the minimum operations needed in order to create respective instruments. The study presented in this manuscript intends to measure the mental effort when creating mobile data collection applications, it was of utmost importance to hold the complexity for all modeling tasks constant. Tasks in divergent complexity, in turn, may limit the validity of the study results as a change in performance measures may be attributed to a more complex model itself or respective mental effort. The overall complexity includes, on one hand, the complexity of the textual representation handed out to the participants and, on the other, the complexity of the resulting data collection instrument. Furthermore, each model contained 2 decision points allowing to change the data collection procedure based on already given answers.

### 2.8. Questionnaires

Throughout the course of the study (see Figure 2), participants had to fill in several questionnaires.

In Session 1, a demographic questionnaire was presented to the participants to collect personal details, like their gender or current field of study. More specifically, prior knowledge regarding *process modeling* was assessed, as this information was later used to separate participants into groups of *novices* and *experts*. After finishing each task, the participants had to answer a *Self-Assessment* questionnaire comprising 5 questions. These questions, in turn, deal with the mental effort of participants when modeling the respective instrument. Thereby, each question provided a 7 point Likert-scale with respective answers ranging from *“I strongly agree”* (1) to *“I strongly disagree”* (7) with an additional *“neutral”* element (4). Due to the fact that questions were phrased in a positive as well as a negative way, the scales were inverted for specific questions. For example, a higher value for the question asking about the mental effort when creating the questionnaire indicate less cognitive load. At the end of both sessions, a short questionnaire assessing the participants own perception regarding the quality of their modeled instruments was presented. In addition, they had to answer if they feel competent in reading models created with the configurator.

In Session 2, only the *Self-Assessment* and *Quality of Models* questionnaires were handed out to the participants.

In the paper at hand, we specifically focus on Question *ME1* of the *Self-Assessment* questionnaire that was filled in after each modeling task. In turn, the question asked was *“The mental effort for modeling the task was considerably high.”*. The participants were allowed to answer with *“I strongly agree”* (1), *“I agree”* (2), *“I rather agree”* (3), *“neutral”* (4), *“I rather disagree”* (5), *“I disagree”* (6), or *“I strongly disagree”* (7).

### 2.9. Statistics

SPSS 25 was used for the statistical analyses. Frequencies (n), percentages (%), means (M), and standard deviations (SD) were calculated for the sample description. Novices and experts were compared in baseline variables via *t*-tests for independent samples and Fisher’s Exact Tests (FET). For Research Question 1, a repeated measures ANOVA with Greenhouse–Geisser correction was applied. Greenhouse–Geisser is a statistical method of adjusting for lack of sphericity. The repeated measures ANOVA had one within-subject factor with 10 levels (10 data collection instruments) and self-rated mental effort (ME) as dependent variable. To address Research Question 2, Pearson correlation coefficients were calculated between the subjective mental effort and the performance measures (i.e., required operations and time as well as made errors) for each data collection instrument. These correlations were calculated separately for novices and experts. To address Research Question 3, linear multilevel models with two levels (Level 1: data collection instruments; Level 2: participants) were performed. The performance measures were the dependent variables in these models and the subjective mental effort was added as predictor. The intercept of the models indicates the performance measure when statistically controlling for the self-reported mental effort (ME). The influence of the predictor mental effort (ME) indicates how the performance measure changes when the subjective mental effort changes one point on the mental effort scale. In addition, standard errors (SE) of each estimate, i.e., standard deviation of its sampling distribution, are reported in the multilevel models. As noted above, higher values stand for *less* mental effort on the used scale. All statistical tests were performed two-tailed and the significance value was set to p<0.05. A *p*-value of <0.05 means that, based on the observed data, the null hypothesis (no correlation, no difference, etc.) can be rejected with a <5% error-probability.

### 2.10. Data Availability

The raw data set containing all collected data that was analyzed during this study is included in this published article (and its supplementary material).

## 3. Results

As described in the study design (see Section 2.2), participants were divided into two groups (i.e., *novices* and *experts*, respectively) based on their prior process modeling experience. Table 3 compares these two samples in baseline variables. The novices sample was larger (45 participants in Session 1; 44 in Session 2) than the experts sample (35 participants in Session 1; 33 in Session 2). The novices sample contained more female participants, whereas the experts sample contained more male participants (p<0.05). In general, the experts sample had more participants with bachelor as highest education level than the novices sample. The novices, however, had a larger amount with high school graduates (p<0.05). Finally, these samples also differ in their field of study; the vast majority of novices studied psychology, whereas the vast majority of experts studied economics or computer science (p<0.05).

### 3.1. Results for RQ 1

How does the self-reported mental effort change when modeling several data collection instruments?

**Figure 4:**
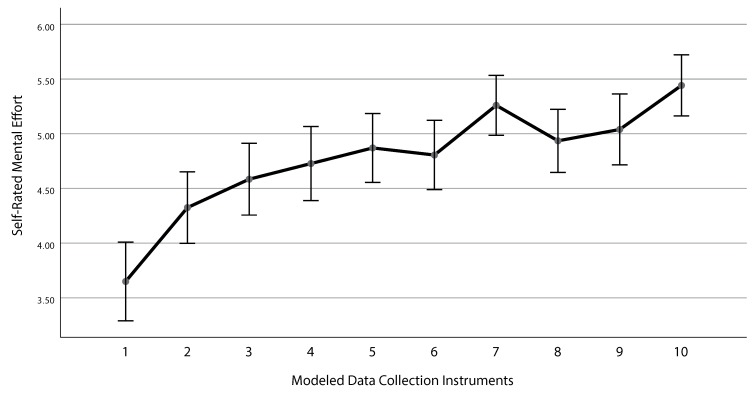
Mean ± 95% confidence interval of the mental effort after modeling data collection instruments.

The repeated measures ANOVA showed a significant result when applying Greenhouse–Geisser correction (F(5.39;76)=21.83; p<0.001). As can be seen in Figure 4, the values of the mental effort increased when more data collection instruments were modeled. This means that the mental effort decreased as higher values indicate less mental effort. Only participants modeling all data collection instruments (N=77) were analyzed.

### 3.2. Results for RQ 2

How are performance measures of novices and experts compared to the self-reported mental effort at each data collection instrument?

In the novices’ sample, mental effort correlated significantly with the performance measures 19 times, whereas mental effort correlated significantly with the performance measures 10 times in the experts’ samples (see Table 4).

### 3.3. Results for RQ 3

How are performance measures of novices and experts compared to the self-reported mental effort across all data collection instruments?

Results for the calculated multilevel models are described in Table 5.

## 4. Discussion

In a prior study, it was revealed that the QuestionSys approach can be efficiently used by non-programmers [48], whereas the study presented herein evaluated the QuestionSys configurator with respect to the mental effort when working with the application. Thereby, 80 participants that were divided into *novices* and *experts*, depending on their prior knowledge in *process modeling*, took part. Over the course of the two sessions of the study, each participant modeled 10 data collection instruments in total. After completing a task, participants had to answer a short questionnaire asking about their mental effort when working with the application.

When evaluating the research questions, results for RQ 1 showed an increase for value of self-report mental effort during the modeling of several data collection instruments, meaning less mental effort. Therefore, the participants required less mental effort the more data collection instruments were modeled. This can be also seen in the assessed performance measures (i.e., time, operations, and errors). Results for RQ 2 indicate a strong correlation of performance measures and mental effort for each data collection instrument. In detail, the novices sample showed 19 (out of 30) significant correlations. By contrast, results from the experts sample show a similar effect, but not in the same extent as described for novices (11 out of 30 significant correlations). This may be explained by the fact that the experts initially assessed the mental effort for creating data collection instruments lower than novices did. Furthermore, their overall performance when creating data collection instruments using the configurator component was better (e.g., they made less errors and were faster) compared to novices. Although this correlation is not surprising, with respect to the concrete results of the correlation, it shows that QuestionSys can be considered perceived comfortably when being used on a day-by-day basis. Regarding RQ 3, a lower mental effort (i.e., indicated by a higher value on the ME scale) correlated with better performance (e.g., less errors) across all data collection instruments. Thereby, both novices and experts showed a significant correlation between the assessed mental effort and the measured performance metrics *time*, *operations*, and *errors*. This effect, however, is stronger for novices. A possible explanation may be that the *experience gain* (i.e., learning effect) is stronger for novices than for experts when continuously working with the developed application over time. In this context, experts are more likely to work with this type of application on a *day-to-day* basis than novices, already having some basic expertise.

Our study carefully considered *external*, *internal*, *construct*, and *conclusion validity* as discussed in [49]. However, some limitations must be discussed. First, the participants involved in the study were mainly students and research associates from Ulm University. Although it is discussed that students may act as proper substitutes in empirical studies [50], another study needs to be carried out in order to confirm the findings of this one. This also applies to the fact that the participants were all <35 years old. It may be interesting to replicate the study with another focus group (e.g., older healthcare professionals). The categorization of recruited participants in *novices* and *experts* based on a single *yes/no* question in the demographic questionnaire may be subject for discussion as well. A more sophisticated categorization may be applied in future research (e.g., by directly observing the individuals when modeling data collection instruments). Along with the participant group, baseline differences between novices and experts could be noticed. The developed configurator, in turn, shall be used by non-programmers (i.e., individuals most likely from non-technical fields of study like social sciences or psychology). Differences in the *field of study* are, therefore, intended in the context of the conducted study. However, both groups did not differ in tests measuring processing speed indicating a similar cognitive ability. Furthermore, the novices sample was slightly larger than the experts one, resulting in a higher statistical power for tests. Finally, the data collection instruments to be modeled may also act as limitation, as some of the participants may be familiar with these scenarios. Likewise, these tasks are from various domains (see Table 2) and did not include modeling external sensors connected to smart mobile devices (e.g., to collect vital parameters). To deal with this limitation, another study specifically focusing on these issues is planned.

In summary, this study replicates results from the previous conducted pilot study in a much broader scope [26]. More importantly, it investigates the self-reported mental effort of participants when working with the developed configurator. The mental effort results, in turn, were compared to collected performance measures (e.g., the time and operations needed to complete respective tasks or the made errors) of the configurator. For novices as well as experts it could be shown that the correlation between self-reported mental effort and all measured performance metrics was significant across all created data collection instruments (i.e., Task 1 to 10). New insights may be gathered when focusing on additional data collected from the *Self-Assessment* questionnaires that were filled in after each of the 10 modeling tasks.

Altogether, when considering the drawbacks of traditional paper-based questionnaires, the QuestionSys configurator may enable researchers from different disciplines to develop instruments themselves. The simplified (graphical) notation thereby fosters the communication between researchers and mobile application developers. Especially large-scale scenarios like clinical trials, in which instruments need to be frequently adapted to new requirements, may significantly benefit from the QuestionSys approach. Moreover, the results show that the mental effort to create such instruments significantly decreases over the time of the study (two sessions with ~1h of modeling). Therefore, the used graphical approach in the developed configurator to create and configure instruments may act as a more general benchmark for mobile data collection procedures in general.

## Figures and Tables

**Table 1:** Realized mobile data collection applications.

Data Collection Scenario	Country	CN	Duration	Versions	Processed Instruments
Study on Tinnitus Research [28]	World-Wide	∘	5 +	5	≥45,000
Risk Factors during Pregnancy [29]	Germany	∘	5 +	5	≥1500
Risk Factors after Pregnancy	Germany	∘	2 +	1	≥500
Posttraumatic Stress Disorder in War Regions [30]	Burundi	•	4 +	5	≥2200
Posttraumatic Stress Disorder in War Regions [31]	Uganda	∘	1 +	1	≥200
Adverse Childhood Experiences [32]	Germany	•	2 +	3	≥150
Learning Deficits among Medical Students	Germany	•	1 +	3	≥200
Supporting Parents after Accidents of Children	EU	∘	3 +	6	≥5000
**Overall**				29	≥54,750
CN = Complex Navigation					

**Table 2:** Short description of tasks to be modeled by participants.

#	Modeling a Questionnaire …	Pages	Decisions
1	…to collect information about flight passengers.	5	2
2	…to help customers selecting an appropriate smartphone.	5	2
3	…to help collecting required information for travel expense reports.	5	2
4	…to order food and drinks online.	5	2
5	…to support customers selecting a movie and booking cinema tickets.	5	2
6	…to help customers selecting an appropriate laptop computer.	5	2
7	…to support customers book seats for a theater play.	5	2
8	…to inform patients regarding their upcoming surgery.	5	2
9	…to guide customers through the process of purchasing a new coffee machine and equipment.	5	2
10	…to collect required data to conclude a contract in a gym.	5	2

**Table 3:** Sample description and comparisons between novices and experts in baseline variables.

Variable	Novices (*N* = 45)	Experts (*N* = 35)	Significance Value
Gender n (%)			p=0.003 (FET)
female	31 (68.9)	12 (34.3)	
male	14 (31.1)	23 (65.7)	
Age n (%)	21.20 (2.63)	22.72 (2.97)	p=0.180 (FET)
<25 years	29 (64.4)	17 (48.6)	
25–35 years	16 (35.6)	18 (51.4)	
Highest Education n (%)			p=0.009 (FET)
High School	13 (28.9)	2 (5.7)	
Bachelor	32 (71.1)	32 (91.4)	
Master	0 (0.0)	1 (2.9)	
Current Field of Study n (%) #			p=0.001 (FET)
Economics	14 (32.6)	12 (40.0)	
Media Computer Science	0 (0.0)	8 (26.7)	
Computer Science	1 (2.3)	6 (20.0)	
International Business	0 (0.0)	1 (3.3)	
Chemistry	2 (4.7)	0 (0.0)	
Psychology	26 (60.5)	3 (10.0)	
Processing Speed Test 1: Digit Symbol-Coding			
Correct Answers M (SD)	84.33 (21.76)	81.11 (21.89)	p=0.515
Wrong Answers M (SD)	0.07 (0.25)	0.06 (0.24)	p=0.864
Processing Speed Test 2: Symbol-Search			
Correct Answers M (SD)	41.93 (7.77)	38.91 (8.53)	p=0.103
Wrong Answers M (SD)	1.73 (1.98)	1.63 (1.50)	p=0.795

Note: FET = Fisher’s Exact Test. # n = 73 of *N* = 80 participants (91%) gave information on their current field of study.

**Table 4:** Correlations between mental effort and performance measures for novices and experts.

			Novices			Experts		
T	S		Operations	Time	Errors	Operations	Time	Errors
1	1	Mental Effort (higher values indicate less mental effort)	−0.126	−0.213	−0.345 *	−0.290	−0.336 *	−0.389 *
2	1		−0.254	−0.289	−0.360 *	−0.434 **	−0.483 **	−0.276
3	1		−0.235	−0.209	−0.303 *	−0.213	−0.42 *	−0.091
4	1		−0.326 *	−0.326 *	−0.478 *	−0.361 *	−0.288	0.043
5	1		−0.083	0.022	−0.379 *	−0.132	−0.082	−0.213
6	2		−0.344 *	−0.273	−0.294	−0.356 *	−0.100	−0.125
7	2		−0.581 **	−0.654 **	−0.395 **	0.078	−0.139	0.048
8	2		−0.575 **	−0.271	−0.382*	−0.109	−0.245	−0.051
9	2		−0.527 **	−0.532 **	−0.369 *	−0.233	−0.426 *	−0.112
10	2		−0.767 **	−0.678 **	−0.332 *	−0.360 *	−0.105	−0.446 **

T = Task; S = Session; with * =p<0.05 and ** =p<0.01.

**Table 5:** Estimates of the multilevel model.

		Parameter	Estimate	SE	df	t	*p*
**Operations**	**Novices**	Intercept	20.26	0.86	445	23.60	<0.001
		ME	−1.64	0.18	445	−9.01	<0.001
	**Experts**	Intercept	20.02	1.26	340	15.86	<0.001
		ME	−1.55	0.24	340	−6.51	<0.001
**Time**	**Novices**	Intercept	399,922.55	22,369.82	445	17.88	<0.001
		ME	−43,497.32	4749.41	445	−9.16	<0.001
	**Experts**	Intercept	402,457.16	31,110.13	340	12.94	<0.001
		ME	−42,536.92	5884.83	340	−7.23	<0.001
**Errors**	**Novices**	Intercept	2.92	0.24	445	12.17	<0.001
		ME	−0.43	0.05	445	−8.53	<0.001
	**Experts**	Intercept	0.88	0.17	335	5.25	<0.001
		ME	−0.11	0.03	335	−3.50	<0.001

SE = Standard Error; ME = Self-reported Mental Effort (higher value = less mental effort); df = Degree of Freedom.

## Supplementary Materials

Supplementary File 1
